# The case for transitional services and programs for older adults reentering society: a narrative review of US departments of correction and recommendations

**DOI:** 10.1108/IJPH-08-2021-0073

**Published:** 2023-02-13

**Authors:** Rose Onyeali, Benjamin A. Howell, D. Keith McInnes, Amanda Emerson, Monica E. Williams

**Affiliations:** Leonard Davis Institute, University of Pennsylvania, Philadelphia, Pennsylvania, USA and is a Clinical Assistant Professor at Geriatric Department, University of Pennsylvania, Philadelphia, Pennsylvania, USA; SEICHE Center for Health and Justice, Yale School of Medicine, New Haven, Connecticut, USA; Center for Healthcare Organization and Implementation Research, VA Bedford Healthcare System, Bedford, Massachusetts, USA and Department of Health Law, Policy and Management, Boston University School of Public Health, Boston, Massachusetts, USA; School of Nursing and Health Studies, University of Missouri-Kansas City, Kansas City, Missouri, USA; Center for the Study of Aging, Rand Corporation, Arlington, Virginia, USA

**Keywords:** Older adults, Health care, Reentry, Prison programs, Prison policies, Transitional healthcare, Aging inmates, Incarceration, Medical services, Graying

## Abstract

**Purpose:**

Older adults who are or have been incarcerated constitute a growing population in the USA. The complex health needs of this group are often inadequately addressed during incarceration and equally so when transitioning back to the community. The purpose of this paper is to discuss the literature on challenges older adults (age 50 and over) face in maintaining health and accessing social services to support health after an incarceration and to outline recommendations to address the most urgent of these needs.

**Design/methodology/approach:**

This study conducted a narrative literature review to identify the complex health conditions and health services needs of incarcerated older adults in the USA and outline three primary barriers they face in accessing health care and social services during reentry.

**Findings:**

Challenges to healthy reentry of older adults include continuity of health care; housing availability; and access to health insurance, disability and other support. The authors recommend policy changes to improve uniformity of care, development of support networks and increased funding to ensure that older adults reentering communities have access to resources necessary to safeguard their health and safety.

**Originality/value:**

This review presents a broad perspective of the current literature on barriers to healthy reentry for older adults in the USA and offers valuable system, program and policy recommendations to address those barriers.

## Background

In 2019, over 1.2 million persons were incarcerated in the US prisons and another 600,000 detained in jails on any given day (Sawyer and Wagner, 2020). An increasing proportion are older adults, defined by the National Commission on Correctional Healthcare as people aged 55 or older (Anno *et al.*, 2004) and by others as 50 or older (Chiu, 2010; DiTomas *et al.*, 2022). Between 1993 and 2013, the older adult population in state prisons increased 400% nationwide, with 131,500 persons over age 55 held in the state prison systems alone in 2013 (Bronson and Carson, 2019; Hawks *et al.*, 2020). Incarcerated older adults represented 30% of the overall prison population (National Corrections Reporting Program, 2000–2012, 2014), similar to their overall percentage in the US population in 2013. The growth in the proportion of imprisoned older adults reflects increases in age of individuals already incarcerated, increasing rates in the incarceration of older persons and longer sentences (Rikard and Rosenberg, 2007; Williams and Rikard, 2004). People over 55 years constitute approximately 12% of people sentenced to more than one year in prison (Bronson and Carson, 2019).

In this article, we describe the literature on the complex health needs of incarcerated older adults and the reentry barriers that confront them as they return to their families and communities following incarceration. While reentry after a jail admission might present many of the same barriers – and some amplified ones – we concentrate on the transition from longer term sentences of a prison incarceration. We focus on older adults’ needs during reentry in discharge planning, health-care access, housing, health insurance and Social Security income. In addition, we suggest areas for future research and policy interventions to improve the health outcomes of older adults during reentry.

## Method

This general review was based on informal surveys of the literature and our pooled expertise as clinicians and researchers who regularly work with older adults who are or have been incarcerated. As a group, we identified key categories of focus in ongoing scholarly and policy conversations at the nexus of aging and health care, incarceration and reentry. We divided the topic areas and consulted work we had on hand along with independently conducted literature searches in PubMed, Scopus and Ebsco databases to identify current emphases in research and policy. We conducted searches of the literature based on key terms reflective of each subtopic area. General or narrative reviews of a topic may be systematic or non-systematic, the latter involving comparatively informal methods of search and selection, in keeping with the form’s emphasis on broad topic overview rather than a systematic evaluation of all available literature to answer a specific question (Ferrari, 2015). Narrative reviews are appropriate for describing general debates and multiple, interrelated questions related to a topic and to speculate on future needs, as was our intent here. In weekly meetings over six months, we discussed our findings, shared sources and gave feedback. The resulting overview addressed:

- the complexity of health and social services needs of older adults while incarcerated and during reentry;
- the challenge of finding housing during reentry; and
- the barriers faced by re-entering older adults in obtaining health insurance and other support to pay for health-care post incarceration.

We defined each area, discussed the literature and identified gaps and opportunities for additional investigation or action.

## Findings

### Health care for older adults during incarceration

Older incarcerated adults face health challenges arising from processes, policies and an environment not designed to meet the needs of persons while aging (DiTomas *et al.*, 2022; Hill, Williams *et al.*, 2006; Zeng and Minton, 2021). Adults with criminal-legal involvement report high rates of chronic disease, including cardiovascular disease, metabolic disease, cancer and chronic obstructive pulmonary disease (Colsher *et al.*, 1992; Binswanger *et al.*, 2009; Maruschak *et al.*, 2015; Wilper *et al.*, 2009; Williams *et al.*, 2012). Of imprisoned adults aged 55 and older, 46.7% report three or more chronic illnesses requiring pharmacological treatment with an average of 7.3 different classes of medication (Williams *et al.*, 2012). Incarcerated adults also report more infectious diseases than community samples, including tuberculosis, human immunodeficiency virus (HIV) and hepatitis (Binswanger *et al.*, 2009; Wilper *et al.*, 2009). Chronic diseases are related to functional impairments in many incarcerated older adults, and the greater the number, the greater the odds of physical impairment (Gates *et al.*, 2018). At the same time, older adults find little accommodations for impairments of vision, hearing, and movement in a setting where the overwhelming priority is security. (Hill *et al.*, 2006). Functional impairments are more prevalent among incarcerated older adults, even after controlling for socioeconomic factors (Greene *et al.*, 2018).

Biological sex modifies the effect of age on the health of incarcerated people (Golembeski *et al.*, 2020). Women who are incarcerated present with aging health concerns earlier than males and at higher costs (Aday and Farney, 2014; Krabill and Aday, 2005; Aday and Krabill, 2006; Lane *et al*., 2020; Rikard and Rosenberg, 2007; Williams and Rikard, 2004).

The cognitive and mental health conditions affecting many older adults may be especially difficult to manage in the carceral setting, where older adults are isolated from family and other social support and exposed to unwanted social contact and even victimization by other incarcerated persons (DiTomas *et al.*, 2022; Hill *et al.*, 2006; Lemieux *et al.*, 2002). An estimated 78% of jail-incarcerated individuals, aged 59–80, in one study, showed mild cognitive impairment on the Montreal Cognitive Assessment (Ahalt *et al.*, 2018) compared to rates of 6.7%–14.8% in adults aged 60–79 in the general population (Petersen *et al.*, 2018).

Time spent in incarceration accelerates and intensifies health issues that the criminal-legal system is ill-equipped to address (Krabill and Aday, 2005; Aday and Krabill, 2006; Lane *et al*., 2020; Anno *et al.*, 2004; DiTomas *et al.*, 2022). Older adults with a history of incarceration show signs of aging and onset of medical conditions approximately 10 years earlier than the general population (Krabill and Aday, 2005; Aday and Krabill, 2006; Lane *et al*., 2020; *Managing Aging and Terminally Ill Inmates*, 2001; Rikard and Rosenberg, 2007). In one analysis of medical problems experienced by older (mean of 59 years) formerly incarcerated individuals, participants reported high prevalence of poor health (52%), chronic lung disease (16%) and recent falls (30%) (Williams *et al*, 2012). In a study of older, low-income, community-living adults (mean of 71.7 years), the prevalence of poor health (51%), chronic lung disease (23%) and recent falls (22%) were similar to those in the younger formerly incarcerated individuals (Counsell *et al.*, 2007). Furthermore, incarcerated women have more complex physical and mental health histories and may exhibit signs of aging 5–10 years earlier than incarcerated men – as early as age 40 or 45 (Krabill and Aday, 2005; Aday and Nation, 2001; *Managing Aging and Terminally Ill Inmates*, 2001; Williams and Rikard, 2004).

Jails and prisons are highly heterogeneous facilities with high turnover of incarcerated individuals (Prison Health Care Costs and Quality, 2017; Sawyer and Wagner, 2020), making generalization about their health-care provision challenging. Some facilities administer their own medical and nursing services, while others contract with local health departments, health systems, medical services corporations and individual clinicians (Prison Health Care Costs and Quality, 2017). Prisons typically offer a broader scope of health services than jails, especially those administered by the Federal Bureau of Prisons (BOP), which follow published clinical guidelines and are organized around a tiered system of care (*Care Level Classification for Medical and Mental Health Conditions or Disabilities*, 2019). Beyond the BOP facilities, there is no federal accreditation requirement or mandated standards for health care at any level of corrections (Stern *et al.*, 2010). While some state-run prison systems and larger jails attain accreditation through the American Correctional Association or the National Commission on Correctional Health Care, it is rarely required by statute, and there are no means outside litigation to ensure standards are met.

## Long-term care while incarcerated

Long-term care for older adults in US prisons is of increasing policy, administrative, legal and humanistic concern. As prison populations age and prisons strain to meet their complex health needs, the costs of staffing, equipment and space increase as well. Health-care costs for an older adult in prison are estimated at $70,000 per year, twice the cost of younger adults (McCarthy, 2013). We identified three key facets of long-term care for this population: access to and management of long-term care in prisons, the practice of compassionate release and access to and management of long-term care upon reentry.

Some facilities use age segregation, the separation of older inmates from the general prison population, to facilitate the delivery of specialized and long-term health services (Wangmo *et al.*, 2017). Proponents argue that age-segregation safeguards the health and wellbeing of older adults and controls costs (Wangmo *et al.*, 2017). Opponents argue that it limits older adults’ federally mandated access to facilities and programs and decrease the possibility of mutually beneficial relationships between older and younger people during an incarceration (Kerbs *et al.*, 2015). Approaches to age segregation include age-specific cell blocks or housing units – in several cases, specifically for older adults with dementia – dedicated facilities for medically complicated individuals and those with daily skilled nursing needs; and contracts with private long-term care facilities or nursing homes (McCarthy, 2013; Vestal, 2014).

Some prisons have instituted programs to encourage self-management of conditions while aging (Williams and Rikard, 2004; Rikard and Rosenberg, 2007). Such programs may provide health and nutrition education, physical activity, art programming, support groups and other social services (Canada *et al.*, 2020; Williams and Rikard, 2004). Proponents of these programs, which are often implemented with community volunteers or other incarcerated persons, cite benefits such as decreasing strain on prison resources and providing more agency for older adults as they age in prison (Harrison and Benedetti, 2009). While these cannot substitute for adequate long-term care, they can complement the care of older adults while not overtaxing already-strained prison resources.

## Compassionate release

Prisons may provide end-of-life and related services for incarcerated older adults with terminal illness who need palliative care or who are nearing death and need hospice care. Compassionate release is the practice of releasing terminally ill or older adult inmates into society to ease the effects of dying, many which are exacerbated by incarceration. To qualify for compassionate release, a candidate must typically no longer be a threat to others (Johns *et al.*, 2021), be unlikely to experience substantial improvement to their mental or physical condition with conventional treatments and must have served at least half their sentences (Hurwitz, 2019). The number of inmates considered for compassionate release is limited by stringent criteria, and even fewer inmates receive approval. Many people die in prison during the cumbersome and lengthy application process (Price, 2018). For persons granted approval, compassionate release may allow them to spend their remaining months or days with loved ones.

### Health-care challenges during reentry and beyond

Discharge planning and transitions of care for people reentering the community after incarceration vary among jails and state and federal prisons, creating significant medical challenges associated with high rates of morbidity and mortality (Binswanger *et al.*, 2009). These processes are particularly difficult for incarcerated older adults with medical comorbidities, cognitive or functional impairment or social support needs. Impairments of vision, hearing, balance, continence and mobility may limit older adults’ ability to obtain documents, apply and coordinate benefits, schedule health care, secure transportation and access medication and other therapies (Gates *et al.*, 2018; DiTomas *et al.*, 2022). Of particular concern for recidivism is cognitive impairment, which doubles the risk of rearrest within six months for older individuals (45% versus 21% for non-cognitively impaired) (Ahalt *et al.*, 2018).

While some services to facilitate reentry exist, many programs are disease- or condition-specific (e.g. for HIV, hepatitis C, substance use disorder) or limited by location. Many older adults must rely on family, faith-based organizations or limited facility- or location-specific resources to provide piecemeal assistance (Boucher *et al.*, 2021). For older adults with complex acute and chronic health-care needs, the informal safety net can be woefully inadequate.

## Acute care

Acute care is a costly component of the health-care system, and overutilization of emergency and urgent care contributes to unnecessary health-care costs. People with a history of incarceration have higher rates of hospitalization and emergency medicine use compared with the general population. In one study, 4.2% of US adults with past-year criminal-legal system involvement accounted for 7.2% of hospital expenditures and 8.5% of emergent care expenditures (Frank *et al.*, 2014). Another study in Medicare enrollees found significantly higher rates of hospitalization following release from prison compared to matched individuals in the general population (Wang *et al.*, 2013). A lack of health insurance and poor access to primary care increase the use of acute care in this population. A cross-sectional study of 247 incarcerated adults over the age of 55 in San Francisco found that 52% reported three-month pre-detainment acute care use and 47% planned to use the emergency department for medical care immediately after release (Chodos *et al.*, 2014). Because most of these individuals were below the age of 65, they qualified for Medicaid by income criteria, but not Medicare. Medicaid typically has lower reimbursement rates and therefore more limited physician acceptance (Rosenbaum, 2014), which leads to difficulty establishing a relationship with a primary care physician to receive preventive care and other care needed to avoid unnecessary hospitalizations.

### Primary and preventive care post-incarceration

Beyond insurance access barriers, other challenges to accessing primary care exist for older adults reentering the community, especially for older adults with more complex chronic medical needs (Puglisi *et al.*, 2022b). Older adults are often released with either no or limited supply of prescription medications and few are connected to primary care providers in the community prior to release (Shavit *et al.*, 2017). Obtaining medical records for care provided while an individual is incarcerated, important for continuity of care, is also challenging and there is often different electronic medical records systems and no infrastructure to support timely transfer of medical records (Solomon *et al.*, 2014). Despite constitutional mandates for health-care provision while incarcerated, there is no corresponding mandate and often little funding for appropriate medical discharge planning or communication with community primary care providers for older adults with complex medical needs. All of these issues often lead to gaps in care, delayed or missed preventive care, avoidable acute-care utilization and increased morbidity (Maschi *et al.*, 2014).

## Long-term care post-incarceration

Older adults reentering the community with long-term care needs often require assistance in finding, accessing and paying for those services. Medicaid covers some older adults who meet income requirements in some states, but those benefits may be suspended or terminated (depending on the state and the time incarcerated) during incarceration by the Medicaid “inmate exclusion” (Spillman *et al.*, 2017), requiring an application to restart benefits on reentry. Some, but not all, prisons coordinate this process prior to release.

Older adults who return to the community in need of long-term health-care services may access care in licensed and accredited skilled nursing facilities and nursing homes, assisted living programs or adult foster homes, or they may find informal care through family, friends and paid helpers (Boucher *et al.*, 2021). Where they exist at all, processes and systems for coordinating long-term health care for older adults after an incarceration vary. The Medicaid “health home” model, a state-optional Medicaid benefit, was designed to increase access of qualified persons (i.e. enrolled in Medicaid and having two or more chronic conditions) to comprehensive managed care across a range of services, including long-term health care. It has been adopted by 21 states and the District of Columbia (Sanchez Ortiz and Barker, 2021) and can provide care to low-income persons with criminal-legal system involvement (Spillman *et al.*, 2017). In a few states, including Rhode Island and New York, the program specifically facilitates access by persons with criminal-legal system involvement (Spillman *et al.*, 2017). These programs link providers and correctional systems to establish discharge referrals and to ensure continuity of health care during transitions. When older adults with medical and skilled nursing needs are released from prison without sufficient planning or assistance accessing services, dangerous disruptions in care and overburdening of caregivers and communities can result (Jimenez *et al.*, 2021).

## Mortality

People with a recent history of incarceration are at a higher risk of premature death.

Internationally, there is a four-fold increase in age-adjusted mortality within the first year after release compared to the general population (Joukamaa, 1998; Morenoff and Harding, 2014). A study by the Washington State Department of Corrections found a relative risk of death 12.7 times that of other state residents two weeks after release (Binswanger *et al.*, 2007). Those under age 45 died more often from overdose, homicide and suicide, while those over 45 died more often from cardiovascular disease and cancer (Binswanger *et al.*, 2007; Binswanger *et al.*, 2013). This elevated risk of death decreased after two weeks but persisted through greater than 2 years of follow-up by a factor of 3.5 (Binswanger *et al.*, 2007).

## Housing challenges during reentry

Housing is a cornerstone of community integration – providing shelter, privacy and safety for persons following incarceration – and has symbolic value, signaling independence and community belonging that can help reduce the stigma associated with incarceration (Keene *et al.*, 2018). Homelessness or unstable housing after incarceration is associated with negative outcomes, such as higher rates of recidivism (Bradley *et al.*, 2001; Fontaine and Biess, 2012; Lutze *et al.*, 2014; Metraux and Culhane, 2004), hospitalization for acute conditions (Erlyana *et al.*, 2014), emergency department use (Wang *et al.*, 2013) and unemployment (Bradley *et al.*, 2001).

The lack of affordable housing significantly affects persons post-incarceration, many of whom transition back into the community with no income or savings and limited social support (Bradley *et al.*, 2001; Roman and Travis, 2006). While publicly subsidized housing might alleviate the burden somewhat, many persons leaving incarceration find themselves disqualified from housing benefits by their criminal records. Some access subsidized housing indirectly by moving in with family members who receive assistance, a risky strategy that violates terms of eligibility and could jeopardize the family’s voucher access (Keene *et al.*, 2018). Perceived (rather than actual) restrictions may also discourage persons from applying for housing assistance. Reports suggest public housing authorities consciously or subconsciously limit access to subsidized housing for formerly incarcerated persons, instead favoring those they deem more “deserving” (Keene *et al.*, 2018). The lack of options, real or perceived, results in many returning citizens taking shelter with family, friends, acquaintances or strangers in transient and/or unsafe situations where the risk of recidivism may increase (Kubrin and Stewart, 2006; Mears *et al.*, 2008; Morenoff and Harding, 2014; Emerson, 2018).

Most research on housing after incarceration does not stratify by age. The available literature describes a daunting situation for older adults reentering the community. In the Boston Reentry Study, those 44 years of age and older experienced 4.4 times more housing insecurity (living on the streets, staying in a homeless shelter, in a residential treatment program) than those under 30 in the first week post-release (70% versus 16%) (Western, 2015). The difference diminished over time, but persisted even at six months (54% versus 17%). Increasing age is associated with higher likelihood of a shelter stay after incarceration – of those aged 55 and older, 22.6% reported a post-release stay in homelessness shelter, compared with 6.4% of persons aged 18–29 (Metraux and Culhane, 2004). Formerly incarcerated older adults may find themselves in competition with younger counterparts for housing and employment (Eggleston and Laub, 2002; Sampson, 2009). Western *et al.* (2015) noted that older persons reentering the community after imprisonment “were the least socially integrated, with weak family ties, unstable housing and low levels of employment” (p. 1512).

### Access to health insurance and other support: role of stakeholders

Multiple stakeholders share responsibility for facilitating the reentry process. From the criminal legal perspective, reentering older adults move from direct supervision by the prison or jail system to either no supervision or community correctional supervision (parole/probation).

From a health-care provision perspective, they re-enter communities with often fragmented and difficult-to-access health-care provision. Payment for health care can be a particular barrier, because much of that payment shifts on release from the correctional system to Medicare, Medicaid or private health insurance. Many older adults also are eligible for benefits from the Social Security Administration (SSA) such as Social Security Disability Insurance or Supplemental Security Income (SSDI/SSI). Each stakeholder has a role in improving the process of discharge planning. Not only do they have some legal responsibility, but they may bear the cost and consequences of adverse health outcomes of older adults during reentry. Importantly, even though these different stakeholders often work together and have overlapping responsibilities, they also have different priorities. Whereas the priority of criminal-legal systems is to maximize public safety and prevent recidivism, the goal for health-care systems is to maximize health outcomes.

## Correctional systems

The responsibility of planning reentry often falls to correctional systems. Most prison systems have some process of discharge planning, which can include plans for housing, education, employment and health-care access (La Vigne *et al.*, 2008). In some jurisdictions, correctional systems partner with community reentry programs and halfway houses, but the availability and quality of these services varies (Prison Health Care Costs and Quality, 2017). National Commission on Correctional Healthcare standards recommend that discharge planning include programs to ensure that “patient’s health needs are met during transition to community health care professionals,” which can include providing planning around medical appointments with community providers, provision of medications and enrollment in health insurance. For individuals with specific health conditions, such as HIV, there is often more comprehensive reentry planning and service connection. Jails, with shorter periods of incarceration (often on the order of days), typically have fewer resources available for discharge planning.

In many jurisdictions, reentering individuals remain under correctional supervision in either parole or probation. Although they can include programming geared toward rehabilitation, societal reintegration and health care, correctional systems’ priority is continuing correctional sanctions and surveillance. *Estelle* v *Gamble*, the Supreme Court ruling that provision of adequate health care is required in correctional settings, does not address provision of health care for individuals under community supervision (Appelbaum, 2020). It is also unclear whether *Estelle* v *Gamble* covers discharge planning (Appelbaum, 2020; Mellow and Greifinger, 2008). That said, given that access to care and improved health outcomes reduce recidivism, correctional systems can better achieve their public safety goals by adjusting their reentry planning and community supervision to address these needs.

## Medicare and Medicaid

Adults over 65 and those between 55 and 64 with a qualifying condition or disability are eligible for Medicare coverage through Social Security. In addition, in the 39 states that had expanded Medicaid coverage as of February 2022 (Kaiser Family Foundation, 2022), most older adults released from prison or jail were eligible for Medicaid. Of the 12 states that have not approved Medicaid expansion, seven are among the top 12 states for rates of imprisonment – Mississippi, Georgia, Alabama, Wyoming, Texas, Tennessee and South Dakota – where the imprisoned population ranged 824–1,094 per 100,000 of total state population (Widra and Herring, 2021). Older adults in the Medicaid non-expanded states may be at an even greater disadvantage than those in the expanded states because their access to health insurance coverage is that much more limited. In general though, even in states with expanded Medicaid coverage, obstacles are numerous. When older adults leave prison, they are also at higher risk, because the reentry period is associated with increased hospitalization and emergency department use, particularly in the Medicare population (Wang *et al.*, 2013). Therefore, besides having a statutory obligation to improve health-care access for this population, Medicare and Medicaid also have a financial incentive to improve discharge planning.

Efforts to increase the role of Medicaid and Medicare in reentry planning are complicated. The Medicaid Inmate Exclusion Policy (MIEP), first passed in 1965, prohibits payment of most health-care services provided to incarcerated individuals by Medicare or Medicaid (Albertson *et al.*, 2020; Khatri and Winkelman, 2022) and often leads to Medicaid termination or suspension during incarceration. Several efforts, such as via Medicaid 1,115 waivers, have attempted to improve enrollment in Medicaid for incarcerated individuals (Albertson *et al.*, 2020). In addition, there has been advocacy at the federal level to repeal this policy, primarily focusing on allowing re-initiation of Medicaid benefits in the final 30 days of an individual’s sentence (Khatri and Winkelman, 2022).

## Social security

The US SSA, established in the Social Security Act of 1935 and now operated by the Department of Health and Human Services (Social Security Administration, 2023), administers Social Security retirement income, SSI, SSDI programs and some aspects of Medicare including enrollment. Social Security retirement benefits are paid monthly to US citizens as early as age 62, if they have worked 10 years cumulatively and paid Social Security taxes from their income (Social Security Administration, 2022). Along with Medicare (also a Social Security program) Social Security retirement income, SSI and SSDI programs provide income on which many adults over age 65 in the US survive. In 2009, President Obama signed into law H.R. 4218 (i.e. the “No Social Security Benefits for Prisoners Act of 2009”) to exclude citizens from receiving retroactive Social Security benefits for periods when they are incarcerated in prison, fleeing to avoid prosecution for a felony or in violation of a parole or probation (Social Security Administration, 2023).

Currently, monthly Social Security retirement payments are suspended after 30 days of continuous incarceration, beginning once a correctional facility notifies the SSA (Re-entering the Community After Incarceration – How We Can Help, 2021). After 12 months of incarceration, SSI and SSDI benefits are terminated. Resumption of suspended benefits in these programs post-incarceration occurs through notification of the SSA (i.e. proof of release). Some correctional facilities have agreements with the SSA and can directly facilitate the resumption of suspended retirement benefits, SSDI and SSI through pre- release notification. Terminated SSI and SSDI benefits require submission of a new application, which can be a lengthy process requiring submission of medical and other documentation and may be difficult for older adults without assistance. Prisons run by the Federal BOP have clinical guidelines for social workers to reconnect persons with Social Security benefits before reentry, a process that can begin up to 120 days prior to release (Community Release Planning Guidelines for Social Work, 2014). It is unclear what proportion of older adults leaving federal prisons are offered the service. Many jails lack pre-release services altogether and nationally there is no designated entity responsible for administering or standardizing these services at any other than the federal prison level.

Nevertheless, programs have been proposed and trialed to facilitate resumption of or application to receive Social Security benefits prior to release. The SSI/SSDI Outreach, Access, Recovery (SOAR) program, sponsored by the Substance Abuse and Mental Health Administration (SAMHSA), offers online training to professionals from communities to guide persons through the application process, including persons prior to reentry. In one study, SOAR demonstrated a 67% initial SSDI application approval rate (Kauff *et al.*, 2016), 33 percentage points higher than the unassisted rate (Policy Basics: Social Security Disability Insurance, 2020). New York state has a program for persons returning to the community with serious mental illness, and Philadelphia has a program for those reentering with substance use disorders and mental illness. Texas works with some older adults who are eligible for their pre-release application program as well (Conly, 2005). Despite promising results from existing programs, their piecemeal distribution and limited reach leaves many older adults to face bureaucratic challenges alone.

Older adults who depend on Social Security, especially SSDI and SSI, to pay for housing and other needs may face a complex, confusing process of notification and/or reapplication at a time when those needs are most pressing (Dennis *et al.*, 2014; Kaiser-Nyman, 2019). Disability risk is at its highest in persons 50–64 years, and disability rates triple between age 45 and 65 (Policy Basics: Social Security Disability Insurance, 2020). Because Social Security benefits pay at the end of a month and do not resume until the month after incarceration ends, a person who leaves incarceration in the middle of a month will not receive payment until the end of the following month (“Re-entering the Community After Incarceration – How We Can Help”, 2021). Healthy transitions from incarceration require coordinated, timed notifications and supportive case management to ensure that when older adults return to the community they do so in a way that gives them some chance of a safe, healthy return. Statutes that suspend or terminate benefits during incarceration may follow a certain retributive logic. But they overreach when they become themselves a second round of punishment, dumping persons post incarceration into the community with no income, no housing, no health-care coverage and few resources to facilitate the re-establishment of such supports. A rethinking and rehaul of this system is sorely needed, including the repeal of the MIEP. For older adults, who often have both more and more complex health-care needs, the systemic failure to maintain a social environment conducive to successful reentry is unsafe, unhealthy, expensive and morally dubious.

## Discussion

Incarcerated older adults present with complex medical and mental health needs at earlier ages and at higher rates than the general population, and yet they are often poorly served by correctional systems not designed to provide health care for older adults. At release, older adults face myriad barriers to successful reintegration, such as accessing health care and housing, acquiring health insurance and obtaining financial/material support. A range of stakeholders, including correctional systems, community organizations, Medicare/Medicaid and the SSA, share responsibility for supporting reentering older adults, but there is wide variability across jurisdictions in how these systems function and what they provide, especially correctional systems and community organizations. These systems often have disparate goals, differing statutory authority and little experience working together, leading to poor coordination and a vacuum of responsibility for overseeing reentry.

There is a need for increased effort among health services researchers, correctional system administrators and policymakers to reform correctional systems and improve the systems supporting reentering older adults. First, society must rethink the purpose and effect of continued incarceration of older adults, who are at low risk of recidivism and are poorly served by current correctional environments. This could include reform of compassionate release policies to consider functional impairment as a criterion. Parole boards should include medical professionals and should consider the medical status of older adults. Additionally, better systems are needed to address the increased medical and functional needs of older adults during incarceration, including access to geriatricians and other specialists in the care of older adults.

Appropriate specialized services should be available to reentering older adults who are medically frail or have complex medical needs because transitional housing such as halfway homes may be inadequate to meet the needs of older adults with cognitive and other functional impairments. These specialized services should include primary care clinics especially designed for the medical needs of reentering older adults such as the Transitions Clinic model that incorporate community health workers with personal lived experience of incarceration and care management to address the health-related social needs specific to reentry (Puglisi *et al.*, 2022a; Shavit *et al.*, 2017. Finally, there must be better coordination of services between different stakeholders. Programs federally supported through Veterans Affairs, the Centers for Medicare and Medicaid Service and the SSA should be consistently and automatically reactivated prior to release, with processes in place to assist older adults who qualify but have never received benefits.

## Conclusion

The goal of this analysis was to provide an integrated discussion of the challenges of meeting the health-care needs of older adults during and after incarceration. The literature suggests that there are three key areas in which correctional systems could further assist elders’ reintegration into society: facilitating health-care linkages to improve access and continuity of care after incarceration, facilitating housing in reentry and facilitating support to pay for health care after incarceration. Our top three recommendations are as follows:

1. (Facilitate health-care linkages):
  - improve uniformity and comprehensiveness of health care for older incarcerated individuals across prison systems, including accreditation and standards of care; increase transparency regarding standards of care in the incarceration systems; and require discharge planning for older adults;
  - develop and implement systems to improve medical discharge planning, including connecting older adults with community health-care providers prior to release and transfer of medical records accessible to outside health-care facilities and providers to improve continuity of care. Develop a system for tracking health care of formerly incarcerated adults;
  - support primary care clinics and health-focused reentry programs that are designed for the needs of reentering older adults; and
  - develop a national network of nursing homes for older adults who need long-term care post-incarceration, with bridge programs to connect older adults with appropriate services post-incarceration.
2. (Facilitate housing in reentry): Improve federal funding for programs that assist newly released older adults in finding and obtaining safe, affordable and stable housing.
3. (Facilitate support to pay for health care): Advocate for legislation to facilitate the automatic resumption of health-care coverage, including Medicaid and Medicare, after release from prison.

Older adults who reenter the community after incarceration often do so with inadequate support. Many struggle to find health care, housing and the means to pay for the basic means of survival. Programs exist to ease the transition of reentry for older adults by providing more continuity of health care and other supports, but these initiatives are often isolated, fragmented and underfunded, leaving the people who need them most stranded at a moment of increased vulnerability. Although it is clear this is a problem with multiple facets that need to be addressed, we hope this overview, which points to places of particular need, can move the field forward by highlighting and focusing priorities and energizing future discussion.
